# Casgevy: Innovative Medicinal Products Require Innovative Approaches to Regulatory Assessment

**DOI:** 10.3390/pharmaceutics16070906

**Published:** 2024-07-06

**Authors:** Essam Kerwash, John D. Johnston

**Affiliations:** Medicines and Healthcare Products Regulatory Agency (MHRA), 10 South Colonnade, Canary Wharf, London E14 4PU, UK

**Keywords:** transfusion-dependent β-thalassemia, Casgevy, CRISPR-Cas9-mediated gene-editing technology, Toulmin scheme, effect of cause analysis

## Abstract

Casgevy (exa-cel) is an autologous cellular therapy modified ex vivo by a CRISPR-Cas9-mediated gene-editing technology. For Casgevy to be granted the indication in transfusion-dependent β-thalassemia, one single-arm trial was submitted which was not amenable to conventional statistical analysis of ‘effect of cause’. Therefore, an analysis was conducted on the basis of ‘cause of effect’ making use of the scheme described by Toulmin coupled to an analysis of causal inference. Based on the current data within the submitted study: subjects with transfusion-dependent β-thalassemia no longer needed a red blood cell transfusion with a 93-percent probability if and only if administered Casgevy; PNS = 93%. It is acknowledged that unknown elements of safety may yet be revealed by long-term follow-up of recipients of Casgevy. Its durability of efficacy is, at present, also an unknown that may also be ascertained by long-term follow-up of recipients. The limitations of a causal analysis are related to assumptions of the proposed causal structure which may not capture the complexity of the real world. Overall, the claim that Casgevy is indicated to treat people with transfusion-dependent β-thalassemia is considered to be supported by the results of the submitted study; the benefit–risk evaluation of Casgevy is found to be positive.

## 1. Introduction

Transfusion-dependent β-Thalassemia is an inherited form of hemoglobinopathies characterized by reduced formation of β-globin chains and impaired erythropoiesis leading to anemia, hypoxia, iron overload and dependence on blood transfusion [1]. While Transfusion-dependent β-thalassemia is caused by gene mutations which reduce the synthesis of β-globin and result in reduction in adult hemoglobin (HbA). Casgevy (exa-cel) consists of autologous CD34+ human hematopoietic stem and progenitor cells obtained by leukapheresis which are then modified ex vivo by a CRISPR-Cas9-mediated gene edit of the erythroid enhancer region of the BCL11A gene; the product is then administered back to the subject [2], which leads to an increased γ-globin expression and subsequent formation of fetal hemoglobin (HbF) which compensate for the reduction in HbA.

Statistical inference in clinical trials is needed to verify the efficacy and safety of medicinal products. A randomized, blinded, controlled trial is widely accepted as the best design to evaluate the efficacy of a new treatment because randomization reduces bias, balances covariates and permits a valid test of significance. Large numbers of subjects are included in such trials in order to generate robust data on efficacy. There may be circumstances, however, where randomized controlled trials are not feasible or practical owing to ethical considerations and evidence from single-arm trials may be all that can be obtained [3]. In addition, pharmacokinetic studies may not be feasible in the context of an autologous cell product such as Casgevy.

In the claim for Casgevy to be granted the indication in transfusion-dependent β-thalassemia, one single-arm trial was submitted, i.e., the trial was not randomized, blinded or controlled and so was not amenable to conventional statistical analyses of ‘effect of cause’ [4]. In order to assess the studies from a clinical perspective, analysis was therefore conducted on the basis of ‘cause of effect’, i.e., that an outcome is caused by an identified event; this analysis made use of the scheme described by Toulmin [5,6] coupled to an analysis of causal inference [4].

## 2. Materials and Methods

*The Toulmin method* [5,6] (named after the philosopher Stephen Toulmin) is a style of argumentation that breaks arguments down into six component parts: data, warrant, claim, qualifier, rebuttal and backing. A claim is the assertion that authors put before their audience; the data of an argument provide background to the claim; the warrant links the grounds to the claim; the qualifier suggests that there are instances where the claim may not be correct; the rebuttal is an acknowledgement of another valid view of the situation; and backing refers to additional support.

**Figure 1:**
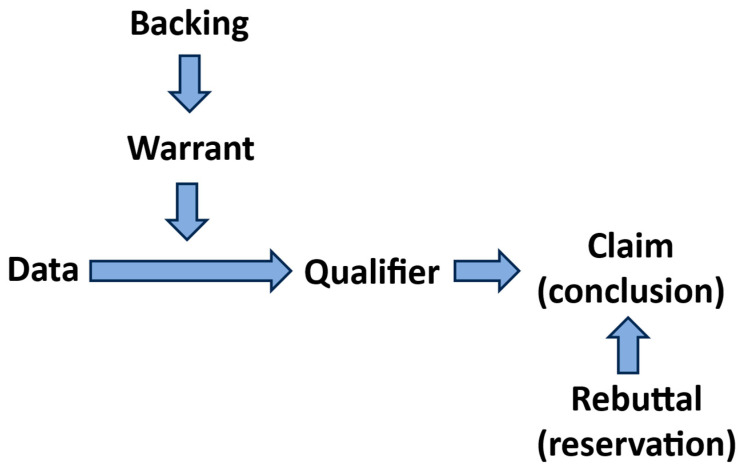
Model of the Toulmin method showing the direction of flow of an argument. The argument takes the form: IF data AND as a result of backed-up warrant THEN claim taking into account qualifier and rebuttal.

Whereas classical argument appeals to a rhetoric analysis of an audience, the Toulmin method (Figure 1) provides an argument in the form of a linear structure where one idea leads to another and so complements a direction-of-flow analysis that is an important component of causal inference.

An understanding of cause requires knowledge of the data-generating process as outlined by Toulmin’s method and coupled to (i) counterfactual analysis, (ii) non-parametric structural equations and (iii) graphical methods.

### 2.1. Graphical Model

**Figure 2:**
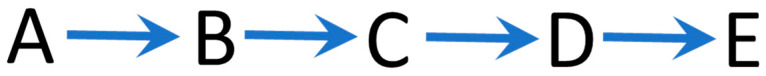
A directed acyclic graph [10] (shown here as a causal chain) representing administration of Casgevy to study participants at A through to the outcome at E. Each node represents an event; each edge points from an earlier time node to a later time node of the same edge. The directed acyclic graph encodes relationships between and amongst variables in the underlying causal structure(s); if, in a graphical model, a variable Y is the child of another variable X, then X is a direct cause of Y. Directed acyclic graphs are non-parametric: they neither specify the form of the causal relationships nor depict the size of the associations and remain qualitative in nature. Letters A to E represent the following: A: Administration of Casgevy (observed and exogenous) to a subject with transfusion-dependent β-thalassemia. B: The CRISPR-Cas9 system introduces a specific break in the binding site of transcription factor GATA1 (so named because the factor binds to a DNA consensus sequence containing G-A-T-A) in the non-coding erythroid lineage-specific enhancer region of the BCL11A gene (that codes for the B-cell lymphoma/leukemia 11A protein) on chromosome 2. All subjects showed an increase in the proportion of alleles with intended genetic modification present in (i) the drug product, (ii) cells in the peripheral blood and (iii) CD34+ cells of the bone marrow [2]; data are consistent with monotonicity. C: Reduction in BCL11A gene transcription in erythroid cells. D: Increase in amount of fetal hemoglobin in red blood cells. All subjects demonstrated an increase in fetal hemoglobin [2]; data are consistent with monotonicity. E for transfusion-dependent β-thalassemia: reduction or abolition of need for red blood cell transfusions. B, C and D are mediators for the effect of A on E. E: The target quantity that can be measured. The model consists of four functions, each representing an autonomous mechanism: b = fb(a, u1), c = fc(b, u2), d = fd(c, u3), e = fe(d, u4). A is a known exogenous variable; the exogenous variables U1–4 represent factors omitted from the analysis. The joint probability distribution is given by P(b,c,d,e|do(a)).

Causal analysis employs directed acyclic graphs to encode causal assumptions. The directed acyclic graph for administration of Casgevy to subjects with transfusion-dependent β-thalassemia is shown in Figure 2 and is made up of an exogenous variable (A), endogenous variables (B,C,D,E) and a set of functions that form a map from A to E.

### 2.2. Counterfactual Analysis and Probabilities of Causation

Pearl [4,7] identifies three counterfactual quantities where causation has two aspects: necessary and sufficient, i.e., some conditions are necessary, meaning that they are needed for something to happen, while other conditions are sufficient, meaning that they are enough to make something happen. Under the assumptions of monotonicity and exogeneity: the probability of necessity (PN), the probability of sufficiency (PS) and the probability of necessity and sufficiency (PNS) may be identified by counterfactual means and may be used to describe the probability that an outcome is attributable to exposure.

Monotonicity expresses the assumption that a change from X = false to X = true cannot, under any circumstance, make Y change from true to false. All medicinal products are exogenous to the recipient. The analysis also relies on the assumption of consistency, i.e., that responses to treatment rely entirely on biological factors and are unaffected by settings where treatment takes place [8,9].

PN, PS and PNS are identified as follows. Let X and Y be two binary variables in a causal model where X is the input and Y is the output; let x and y stand for the propositions X = true and Y = true, respectively; let x′ and y′ be their respective complements.

The probability of necessity (PN) is defined as PN = P(Yx′ = false|X = true, Y = true). In words, PN stands for the probability that event y would not have occurred in the absence of event x, given that x and y did in fact occur. PN is a measure of when something is required for something to happen, i.e., a measure of attribution.

The probability of sufficiency (PS) is defined as PS = P(Yx = true|X = false, Y = false). In words, PS stands for the probability that setting x would produce y in a situation where x and y are in fact absent. PS is a measure of when something is adequate for something to happen.

The probability of necessity and sufficiency (PNS) measures the world in which y is true when x is true and y is false when x is false. PNS is defined as PNS = P(Y = 1|do(X = 1)) − P(Y = 1|do(X = 0). In words, PNS stands for the probability that Y will occur if and only if X has taken place. In the case of transfusion-dependent β-thalassaemia, if Casgevy is not administered, then the probability of abolition of the need for red blood cell transfusions would be zero, i.e., P(Y = 1|do(X = 0) equals 0; PNS then becomes equal to P(Y = 1|do(X = 1)).

## 3. Results

The results section is divided into the following subheadings: data/background information, warrant, backing, qualifier, rebuttal, claim and counterfactual analysis.

The Toulmin scheme for subjects with transfusion-dependent β-thalassemia enrolled by the company is shown:

### 3.1. Data/Background Information

β-thalassemia is an inherited autosomal recessive disorder caused by genetic mutations that reduce or eliminate the expression of β-globin resulting in ineffective erythropoiesis and chronic hemolytic anemia.

People with severe β-thalassemia are dependent upon regular transfusions of red blood cells.

### 3.2. Warrant

This was a single-arm demonstration study that enrolled subjects with transfusion-dependent β-thalassemia.

There were 29 adults (age ≤ 35 yrs) and 13 pediatric subjects (ages ≥ 12 to <18 yrs) in the primary efficacy set at the current interim stage; the median age was 20 yrs; 25/42 subjects had a β0/β0-like genotype, and 30/42 subjects had an intact spleen.

For the primary efficacy set, the median dose of Casgevy was 7.5 (range: 3.0 to 19.7) × 10^6^ CD34+ cells/kg body weight. The median (range) follow-up duration after exa-cel infusion was 23.9 (16.1 to 27.1) months.

For the primary efficacy set: 39/42 subjects achieved the primary outcome by maintaining an average Hb ≥ 9 G/dL without red blood cell transfusions for at least 12 consecutive months any time after exa-cel infusion.

For those who achieved the primary outcome,

- the median time to achieve ‘free from transfusion’ was (about) 1 month and the maximum time was (about) 3 months;
- the total duration of being transfusion-free ranged from (about) 13 to 24 months;
- mean HbF increased from trace concentrations at baseline to 10.8 G/dL by month 6 and remained so for the duration of follow-up.

Adverse events were, in the main, those known to be associated with autologous stem cell transplants and so may be anticipated and managed appropriately.

### 3.3. Backing

Proposed mechanism of action: Exa-cel is introduced into ex vivo autologous hematopoietic stem cells where the CRISPR-Cas9 system creates a targeted break in the DNA of the host cell leading to a cascade effect that results in increased production of fetal hemoglobin. The presence of fetal hemoglobin is associated with a stable form of red blood cell. Engineered stem cells are re-introduced back to the patient.

Other trials: The mechanism of action of exa-cel is the same in patients with transfusion-dependent β-thalassemia and sickle cell disease.

### 3.4. Qualifier

For the 2 years before screening, the baseline median (range) annualized units of thalassemia-related red blood cell transfusions per year was 35.0 (20.5 to 71.0) units, and the baseline median (range) annualized volume of thalassemia-related red blood cell transfusions was 201 (115.2, 330.9) mL/kg per year.

Adverse events associated with Casgevy are, in the main, those associated with the autologous cell transplantation procedure and exposure of subjects to agents that condition the bone marrow prior to administration of Casgevy.

### 3.5. Rebuttal

A single-arm demonstration study has been conducted in the context of a rare disease, where the disease is stable/progressive and with the caveat of the fallacy of human reasoning referred to as post hoc ergo propter hoc (Latin: ‘after this, therefore because of this’). An interim report has been submitted.

Outcomes of this study are likely biased because

- the trial had a single-arm design (i.e., without an internal control);
- the trial relied on comparisons of need for red blood cell transfusion in each patient in the preceding year before exposure to Casgevy.

Long-term maintenance of efficacy beyond 36 months has not yet been established.

Three subjects have not achieved freedom from red blood cell transfusion (yet have achieved >80% reduction in need for red blood cell transfusion).

### 3.6. Claim

Casgevy may be indicated to treat people with transfusion-dependent β-thalassemia.

### 3.7. Counterfactual Analysis

Subjects with transfusion-dependent β-thalassemia enrolled by the company had

an observational component where the need for red blood cell transfusions was recorded retrospectively for two years prior to enrolment;

an experimental component where subjects were administered Casgevy as a one-off exercise and then followed-up.

The natural history of the condition of transfusion-dependent β-thalassemia is for subjects to require frequent transfusions of red blood cells in order to maintain a desired concentration of hemoglobin; remission of the condition is not known to occur within a natural history setting.

For subjects enrolled into this study, the median (range) annualized units of thalassemia-related red blood cell transfusions per year was 35.0 (20.5 to 71.0) units for the 2 years before study entry. None of the subjects were able to stop red blood cell transfusions. If the subjects did not receive Casgevy, then they would not be able to stop the need for red blood cell transfusions, i.e., the percent probability Yx′ = false is equal to 100% for the observational period prior to study entry.

The subjects were administered Casgevy in the experimental section of this study; 39/42 subjects were able to stop red blood transfusions altogether. Given the natural history of transfusion-dependent β-thalassemia and the findings of the observational period, it is then considered that if subjects had not been administered Casgevy, then they would not have been able to stop red blood cell transfusions, i.e., there is a 100-percent probability that administration of Casgevy was a necessary cause of no longer needing regular transfusions of red blood cells. PN = 100%.

A total of 39 subjects out of 42 were able to stop red blood cell transfusions. 39/42 = 93%, i.e., there is a 93-percent probability that administration of Casgevy is sufficient to lead to a situation where red blood cell transfusions are no longer needed to maintain a desired hemoglobin concentration. PS = 93%.

It is noted that all the three patients who were unable to stop red blood cell transfusions had received less than 5.5 million cells per kg body weight; it is considered that the probability of sufficiency would be increased by administration of greater than 5.5 million cells per kg body weight.

Based on current data within the submitted study, subjects with transfusion-dependent β-thalassemia no longer needed a red blood cell transfusion with a 93-percent probability if and only if administered Casgevy. PNS = 93%.

## 4. Discussion

Regulatory submissions usually require the inclusion of extensive quality, non-clinical and clinical pharmacology data.

Pre-clinical data suggest that off-target editing does not occur (or cannot be detected by current methods) in the target populations and that Casgevy carries a low risk of CRISPR-Cas9-mediated genotoxicity.

Conventional pharmacokinetic (PK) studies on absorption, distribution, metabolism and excretion are considered to be not feasible in the context of a cell therapy such as Casgevy. Pharmacodynamic (PD) data and persistence of effect, however, were recorded. Persistence of edited cells was assessed by the mean proportion of alleles with the intended genetic modification in CD34+ cells of bone marrow and peripheral blood; persistence was shown to be maintained at ≥60% of alleles in the cells of both subjects with transfusion-dependent thalassemia and sickle cell disease.

A one single-arm clinical study was submitted in order to support the claims of the company [2]. From a clinical perspective, the submitted study was not randomized, not controlled and not blinded. Inferential statistics may be used to compare the differences between treatment groups; yet, within the setting of one single-arm, non-randomized study, they are unable to provide valid probability statements about the treatment effects, owing to their inability to control for bias. Further, single-arm studies are unable to support assumptions that are supported by randomization. Conventional statistics may be used to analyze single-arm studies in a descriptive manner; yet. this would lose the connection to a causal difference in outcome. In order to address these issues, it has been recommended that conventional statistics are de-emphasized in favor of other forms of description [11].

In order to avoid the difficulties associated with conventional statistics in the absence of randomization, a ‘cause of effect’ analysis [4,12] has been employed that makes use of a structural causal model with three parts: (i) a graphical model, (ii) structural equations and (iii) counterfactual and interventional logic. Graphical models display what one knows about the world; counterfactual argument articulates what one wants to know; and structural equations knit (i) and (ii) together [13].

People with transfusion-dependent β-thalassemia require frequent transfusions of red blood cells and will develop symptoms and consequences of iron overload. For these reasons, the main claim that Casgevy can be used to treat people with transfusion-dependent β-thalassemia would be desirable if those treated no longer needed transfusions of red blood cells. In the context of a single-arm study, a binary outcome (subjects either achieved or did not achieve freedom from the need for red blood cell transfusions) is preferred to a continuous outcome (that may be affected by individual variation) or a time-to-event outcome (where there is not a comparator).

At this stage in product development where 39/42 subjects have become free of the need for transfusions of red blood cells within weeks of administration of Casgevy [2], the evidence is found to be dramatic, credible and accurate. The outcome is unlikely to have occurred spontaneously or to have been influenced by other clinical management or alternative therapies.

Aspects of clinical safety have been found to be related, in the main, to the autologous transplantation procedure; it is acknowledged that unknown elements of safety may yet be revealed by long-term follow-up of recipients of Casgevy. Durability of efficacy is, at present, also an unknown that may also be ascertained by long-term follow-up of recipients.

The limitations of a causal analysis are related to assumptions [7] of the proposed causal structure and mechanisms that are displayed in Figure 2; these assumptions may not capture the complexity of the real world.

Future studies characterizing cellular kinetics may employ techniques such as population modelling [14] or mechanistic PK/PD analysis [15,16] to predict cell kinetics and responses to cell therapies; these techniques have the advantage of accounting for the dynamic interaction between the host and the cell therapy. Quantitative systems pharmacology models have also been used [17,18] to link the mechanism of action to clinical outcome and may offer an additional route to investigate personalized cell therapy.

In summary, we have produced a causal analysis for a single-arm trial submitted to make the claim for the treatment of transfusion-dependent beta-thalassemia (with an additional analysis for sickle cell disease provided in the Supplementary Material). Such ‘cause of effect analysis’ provides an alternative to conventional statistics (frequentist and Bayesian) and, in our opinion, avoids the difficulties of conventional statistics associated with single-arm trials (low power of study, lack of randomization and lack of comparator) that hinder interpretation of outcomes.

## 5. Conclusions

Overall, the claim that Casgevy is indicated to treat people with transfusion-dependent β-thalassemia is considered supported by the results of the submitted study; the benefit–risk evaluation of Casgevy is found to be positive.

## Data Availability

Additional data are available upon request to the authors at the following email address: essam.kerwash@mhra.gov.uk.

## Supplementary Materials

The following supporting information can be downloaded at: https://www.mdpi.com/article/10.3390/pharmaceutics16070906/s1, Supplement to display information on subjects with sickle cell disease who were administered Casgevy.
