# Supplementary material for: Casgevy: Innovative Medicinal Products Require Innovative Approaches to Regulatory Assessment
Source: Pharmaceutics. 2024 Jul 6;16(7):906. doi: 10.3390/pharmaceutics16070906 (PMC11279610; doi:10.3390/pharmaceutics16070906)
Supplement: Supplementary file 1 [file pharmaceutics-16-00906-s001.zip › pharmaceutics-2995705-supplementary.pdf]

# Supplementary Materials: Casgevy: Innovative Medicinal Products Require Innovative Approaches to Regulatory Assessment

**Essam Kerwash and John D. Johnston**

Supplement to display information on subjects with sickle cell disease who were administered Casgevy.

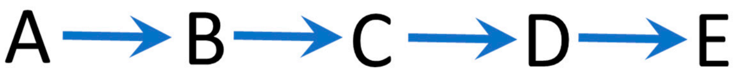


**Figure S1.** Directed acyclic graph (shown here as a causal chain) representing administration of Casgevy to study participants at A through to outcome at E. Each node represents an event; each edge points from an earlier time node to a later time node of the same edge. The directed acyclic graph encodes relationships between and amongst variables in the underlying causal structure(s); if, in a graphical model, a variable Y is the child of another variable X, then X is a direct cause of Y. Directed acyclic graphs are non-parametric: they neither specify the form of the causal relationships nor depict the size of the associations and remain qualitative in nature. Letters A to E represent the following:.

A: Administration of Casgevy (observed and exogenous) to a subject with sickle cell disease.

B: The CRISPR-Cas9 system introduces a specific break in the binding site of transcription factor GATA1 (so named because the factor binds to a DNA consensus sequence containing G-A-T-A) in the non-coding erythroid lineage-specific enhancer region of the BCL11A gene (that codes for the B-cell lymphoma / leukaemia 11A protein) on chromosome 2.

All subjects showed an increase in the proportion of alleles with intended genetic modification present in (i) the drug product, (ii) cells in the peripheral blood and (iii) CD34+ cells of the bone marrow [ref to UKPAR]; data are consistent with monotonicity.

C: Reduction in BCL11A gene transcription in erythroid cells.

D: Increase in amount of fetal haemoglobin in red blood cells.

All subjects demonstrated an increase in fetal haemoglobin [ref to UKPAR]; data are consistent with monotonicity).

E for sickle cell disease: Reduction or abolition of sickle crises.

B, C and D are mediators for the effect of A on E.

E is the target quantity that can be measured.

**Results**

The Toulmin scheme for subjects with sickle cell disease enrolled by the company is shown:

Data / background information: Sickle cell disease is caused by a single-nucleotide substitution in DNA resulting in valine replacing glutamic acid at position 6 of the β-globin chain of haemoglobin. This form of haemoglobin - HbS polymerises in the deoxygenated state causing distortion of the shape of red blood cells; these sickle cells have a reduced lifespan and may clump and block blood vessels resulting in painful crises, organ failure and early mortality. The most common causes of death are cardio-pulmonary, cerebrovascular and renal; there is a high risk of sudden death; the median age at death is 45 years.

Management of sickle cell disease is aimed at avoiding pain episodes, relieving symptoms and preventing complications. Subjects are advised to reduce the chances of a sickle crisis by maintaining hydration and keeping warm.

Warrant: a single-arm demonstration study that enrolled subjects with severe sickle cell disease but are able to undertake normal daily activities.

There are 29 subjects in the primary efficacy set; 45% were male; the median age was 21yrs (min 12yrs, max 34yrs).

Subjects had documented βS/βS, βS/β0 or βS/β+ genotypes and a history of at least two events (acute chest syndrome, priapism, splenic sequestration or attending a medical facility for management of an acute pain event) in the 2 years before the study whilst receiving appropriate supportive care.

For the primary efficacy set: the median (range) dose of Casgevy was 4.0 (2.9 to 14.4) $\times 10^6$ CD34+ cells/kg body weight; the median (range) follow-up duration after exa-cel infusion was 23.6 (16.1 to 25.6) months.

Of the subjects, 28/29 achieved the primary outcome by not experiencing any severe vaso-occlusive crisis for at least 12 consecutive months after exa-cel infusion.

- There were increases in mean total Hb and HbF concentrations for subjects in the primary efficacy set; these increases occurred early (Month 3) and were maintained over time.
- All 29 (100%) subjects in the primary efficacy set sustained HbF ≥20% for at least 12 months.

Adverse events were, in the main, those known to be associated with autologous stem cell transplants and so may be anticipated and managed appropriately.

Backing: Proposed mechanism of action: Exa-cel is introduced into ex vivo autologous haematopoietic stem cells where the CRISPR-Cas9 system creates a targeted break in the DNA of the host cell leading to a cascade effect that results in increased production of fetal haemoglobin. The presence of fetal haemoglobin is associated with a stable form of red blood cell. Engineered stem cells are re-introduced back to the patient.

Other studies: The mechanism of action of exa-cel is the same in patients with transfusion-dependent β-thalassemia and sickle cell disease.

Qualifier: For the 2 years before enrolment in the primary efficacy set, the median annualised rate of severe vaso-occlusive crises was 3 (min 2, max 9.5); the median annualised rate for in-patient management of severe vaso-occlusive crises was 2.0 (min 0.5, max 8.5); the median annualised duration of stay for in-patient management of severe vaso-occlusive crises was 12.5 days (max 65 days); and the annualised number of units of red blood cells transfused for sickle cell disease was 3.5 (min 0, max 75.5).

Adverse events associated with Casgevy are, in the main, those associated with the autologous cell transplantation procedure and exposure of subjects to agents that condition the bone marrow prior to administration of Casgevy.

Rebuttal: A single-arm demonstration study has been conducted in the context of a rare disease, where the disease is stable / progressive and with the caveat of the fallacy of human reasoning referred to as post hoc ergo propter hoc (Latin: 'after this, therefore because of this'). An interim report is submitted.

The outcomes of the study are likely biased because: (i) the trial had a single-arm design (i.e. without an internal control); (ii) the trial relied on comparison to number / frequency of sickle episodes in each patient in the preceding year before exposure to Casgevy; and (iii) the outcome of vaso-occlusive crisis is considered to have a notable subjective component.

Long-term maintenance of efficacy beyond 36 months has not yet been established.

One subject had recurrence of crises that may/may not represent loss of efficacy.

Claim: Casgevy is indicated to treat people with sickle cell disease.

Counterfactual analysis

Subjects with sickle cell disease enrolled by the company had (i) an observational component where the need for clinical management of crises was recorded retrospectively for two years prior to enrolment and (ii) an experimental component where subjects were administered Casgevy as a one-off exercise and then followed-up.

The natural history of the condition of sickle cell disease is for subjects to require frequent interactions with healthcare professionals to manage sickle crises; remission of the condition is not known to occur within a natural history setting.

For subjects with sickle cell disease enrolled into the study, the median annualised rate of severe vaso-occlusive crises was 3 (min 2, max 9.5) for the 2 years before enrolment in the primary efficacy set. None of the subjects were free of crises prior to enrolment. If the subjects did not receive Casgevy, then they would not be able to avoid a crisis, i.e., the percent probability Yx′ = false is equal to 100% for the observational period prior to study entry.

Subjects were administered Casgevy in the experimental section of the study; 28/29 subjects were free of crises after administration of Casgevy; given the natural history of sickle cell disease and the findings of the observational period, then it is considered that if subjects had not been administered Casgevy, then they would not have been able to be free of crises, i.e., there is a 100-percent probability that administration of Casgevy was a necessary cause of being free of crises. PN = 100%.

Of the patients, 28/29 were free of crises after administration of Casgevy. 28/29 = 96%, i.e., there is a 96-percent probability that administration of Casgevy is sufficient to lead to a situation where subjects are free of crises. PS = 96%.

The subject who experienced a crisis after Casgevy administration had received less than 5.5 million cells per kg body weight; with reference to information from the b-thalassaemia subjects, it is considered that the probability of sufficiency would be increased by administration of greater than 5.5 million cells per kg body weight.

Based on current data within the submitted study, subjects with sickle cell disease may become free of crises with a 96-percent probability if and only if administered Casgevy. PNS = 96%.
